# Continuous use of visual information about the position of the moving hand

**DOI:** 10.1007/s00221-023-06658-x

**Published:** 2023-06-29

**Authors:** Eli Brenner, Jeroen B. J. Smeets

**Affiliations:** Department of Human Movement Sciences, Vrije Universiteit Amsterdam, Van der Boechorststraat 7, 1081 BT Amsterdam, The Netherlands

**Keywords:** Online control, Arm movements, Displacements, Ballistic, Human, Proprioception

## Abstract

People generally look at a target when they want to reach for it. Doing so presumably helps them continuously update their judgments about the target’s position and motion. But not looking at their hand does not prevent people from updating judgments about its position on the basis of visual information, because people do respond to experimental perturbations of visual information about the position of their hand. Here, we study such responses by adding jitter to the movement of a cursor that follows participants’ fingers. We analyse the response to the jitter in a way that reveals how the vigour of the response depends on the moment during the movement at which the change in cursor position occurs. We compare the change in vigour to that for equivalent jitter in the position of the target. We find that participants respond to jitter in the position of a cursor in much the same way as they respond to jitter in the target’s position. The responses are more vigorous late in the movement, when adjustments need to be made within less time, but similarly so for the cursor as for the target. The responses are weaker for the cursor, presumably because of the jitter-free kinaesthetic information about the position of the finger.

## Introduction

There is a long history of considering goal-directed arm movements to consist of distinct stages: an initial ballistic stage, followed by a stage in which the movement is controlled on the basis of feedback (Elliott et al. 2017; Woodworth 1899). However, the asymmetric velocity profile that is at the basis of this distinction is easy to explain in terms of optimising control (Scott 2004; Todorov 2004). Moving more slowly near the end of a movement can be useful for guiding the hand precisely to its goal. It also reduces the potential damage caused by errors. Similarly, increasing the vigour of responses to errors as the movement progresses (including ‘errors’ that are artificially imposed by shifting the target) is beneficial, because as the movement progresses, there is less and less time in which to make the necessary adjustment (Brenner et al. 2022, 2023; Oostwoud Wijdenes et al. 2011; Zhang et al. 2018). Consequently, except at the very end of the movement, the vigour of responses to target shifts is inversely related to the remaining time (Brenner et al. 2022). How about visual information about the position of the hand? The same reasoning predicts that the vigour of responses to shifting the hand will increase as the movement progresses, but there are some reasons why visual information about the hand might be treated differently.

When reaching out for objects, people generally look at the object as their hand approaches it (Hayhoe and Ballard 2005; Land 2006; Land and Hayhoe 2001; Neggers and Bekkering 2000; Voudouris et al. 2018). They seldom look at their moving hand, or at a cursor representing the moving hand (Cámara et al. 2020). They seldom even look at a cursor representing the hand when the delay with which the cursor follows the hand varies unpredictably (Cámara et al. 2018). One might be tempted to conclude from the fact that people do not look at their hand that people rely exclusively on the felt position of their hand to guide their movements. However, this cannot be true, because hiding the hand impairs performance (Berkinblit et al. 1995; Carlton 1981) and people quickly adjust their movements when a cursor that follows the hand is unexpectedly displaced (Brenner and Smeets 2003; Saunders and Knill 2003, 2004, 2005; Franklin et al. 2016) or the delay with which it follows the hand varies across movements, so that it is unknown until the cursor starts moving (Cámara et al. 2020). People must therefore use peripheral visual information concerning their hand to guide their movements.

People presumably direct their gaze at the target of the movement, because they must rely on precise (foveal) visual information about the target, whereas they can combine less precise peripheral visual information with kinaesthetic information about the moving hand (Kasuga et al. 2022; Sober and Sabes 2005). If both kinaesthetic information and visual information are used to guide the hand, visual information may be given less weight early in the movement, when the hand (or cursor) is further from the target. The larger distance from the target, and therefore from where one is looking, means that the information is provided at a larger retinal eccentricity, and therefore with a lower resolution. Moreover, early in the movement, kinaesthetic information should be precise enough to guide the hand in approximately the correct direction, whereas later in the movement, it can be important to remove errors that arise from persistent mismatches between the felt and seen position (Kuling et al. 2016; Smeets et al. 2006). If this reasoning is correct, we expect the response to perturbations to increase more strongly for the cursor than for the target as the movement proceeds.

## Methods

**Fig. 1:**
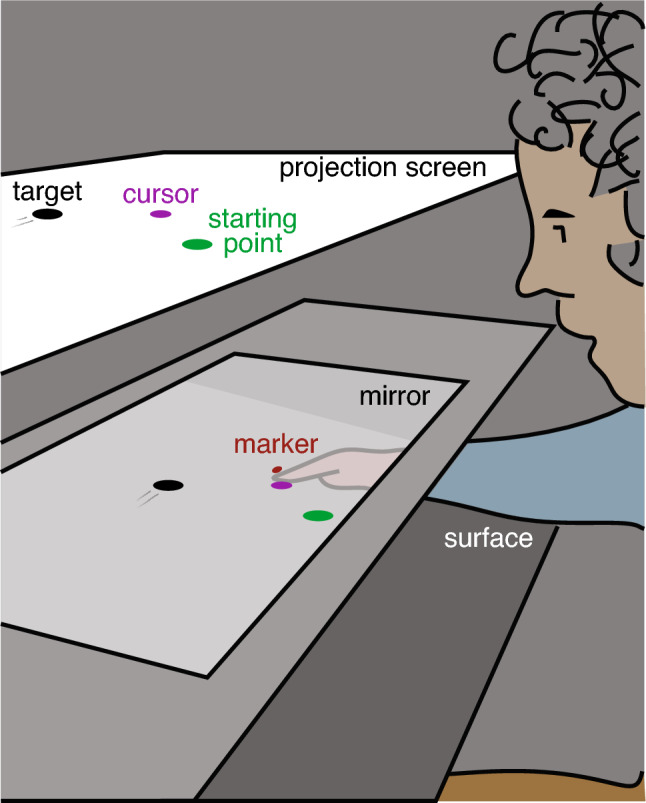
Schematic representation of the set-up. A target, cursor, and starting point were projected from above onto a back-projection screen. The participant looked at the screen from below by looking into a mirror that blocked his or her view of the hand. The distance between the projection screen and the mirror was equal to that between the mirror and the surface across which the finger moved, so the items on the screen appeared to be on that surface. The participant’s task was to slide his or her unseen finger across the surface, such that the cursor passed through the moving target. The cursor followed the position of the marker attached to the fingernail. The starting point and target were never present at the same time

**Fig. 2:**
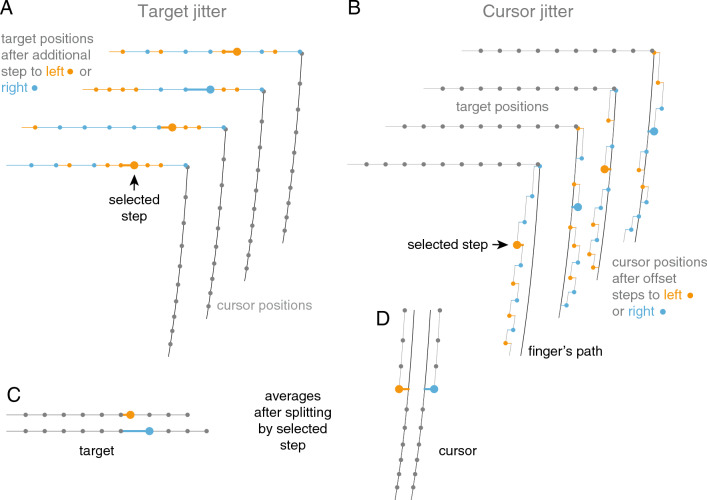
Schematic representation of the stimulus and analysis. Target and cursor positions during the last 167 ms of four trials, with random leftwards and rightwards steps added to the target’s rightward motion (**A**) or to the cursor offset (**B**). The larger dot indicates a selected step (here the fourth before the end). Averaging many trials in which the selected step was either to the left (large orange dots) or to the right (large blue dots) results in a systematic 5 mm difference between the target positions (**C**) or cursor offsets (**D**) from that moment onward

To compare how the vigour of responses to cursor and target perturbations changes during a fast goal-directed movement, we used a method that we recently developed to study responses to target perturbations (Brenner et al. 2023). We asked participants to intercept a moving target with a cursor that followed their occluded finger (Fig. 1). In separate trials, we added small steps to either the target’s lateral position or the cursor’s lateral distance from the finger (cursor offset) on every image frame. Whether each step was to the left or to the right was chosen at random throughout the movement (Fig. 2A, B). We could then separate trials by the direction of the step at specified moments to see how a step at that moment influenced the lateral movement of the finger. By doing this for many moments during the movement, we get an impression of how the vigour changes during the movement. We then compare changes in the vigour of responses to steps in the cursor offset with changes in the vigour of responses to steps in the target position.

The jitter in the motion of the target or cursor that resulted from adding the random steps was hardly visible when the target or cursor was moving. It was visible when the cursor jittered and the hand had not yet started to move. We did not expect whether or not the jitter was visible to influence the responses, because responses to target perturbations do not rely on noticing the perturbation (Goodale et al. 1986; Gritsenko et al. 2009). Moreover, we have already established that the vigour of responses to target jitter does not depend on whether or not the jitter is visible (comparison of responses to jitter of static and moving targets in Brenner et al. 2023). Thus, we do not expect the visibility of the cursor jitter early in the movement to change the relationship between responses to steps in cursor offset and in target position.

### Participants and sessions

Twenty young adults took part in the experiment. Two of them knew the purpose of the experiment. Each participant took part in two similar sessions of 200 trials, separated by a short break. In each session, 100 trials in which the cursor jittered were randomly interleaved with 100 trials in which the target jittered. Six sessions terminated prematurely for technical reasons. In those cases, the participants were asked to take part in additional (shorter) sessions to ensure that all participants had performed at least 200 trials in which the target jittered and 200 trials in which the cursor jittered. As a result, we had slightly more than 400 trials for some participants.

### Equipment and calibration

Participants moved their hand below a mirror (Fig. 1). An infrared light-emitting diode was attached to the nail of the index finger of what the participant reported to be his or her dominant hand. This diode’s position was determined at 500 Hz using an Optotrak 3020 motion capture system. A second diode was used to synchronise the measured positions with the image presentation. It was placed at a fixed position, and its power was turned off, resulting in it being invisible to the Optotrak, whenever light fell on a sensor at the top left corner of the screen. By presenting a flash in that corner whenever a new target appeared, we could determine when targets appeared with respect to the measured finger positions to within 2 ms.

Images were 1024 × 768 pixels (65 × 48 cm), presented at 60 Hz using a Hitachi CP-X325 LCD Projector. To spatially align the measured finger positions with positions on the screen, we asked participants to place their finger on four small targets at the beginning of each session. Taking advantage of the fact that the mirror was half-silvered, we illuminated the area below the mirror to allow participants to see their finger when aligning it with the targets. We then used the four indicated positions to determine the transformation between measured diode positions and the positions on the screen that the finger was aligned with. Doing so compensates for the fact that the diode is attached to the fingernail rather than to the point that the participant aligns with the target. During the rest of the session, the area below the mirror was not illuminated, so the finger was not visible.

### Procedure

Participants started a trial by moving their finger to the starting point, a green, 2 cm-diameter disc. Doing so moved a 1 cm-diameter purple disc (the cursor) to that position. The background was white. If the participant kept his or her finger within the starting point for a randomly selected duration between 0.6 and 1.2 s, the starting point disappeared and the moving target appeared. If the finger left the starting point before the randomly selected duration had expired, the target did not appear and the participant had to move his or her finger back to the starting point.

The target was a 2.7 cm-diameter black disc. It appeared 30 cm to the left of and 20 cm further away from the participant than the starting point. It moved at 50 cm/s to the right, with additional jitter on half of the trials. On the other half of the trials, the cursor started to jitter when the target appeared. The jitter was added to the position of the moving target or to the cursor offset. It consisted of an additional lateral 2.5 mm step at each image presentation (60 Hz), randomly either to the left or to the right. These steps were accumulated in the form of a random walk (see schematic examples in Fig. 2A, B; a 2.5 mm step corresponds with a velocity of 15 cm/s).

The task was to slide the cursor through the target without lifting the finger off the surface. If the (interpolated) centre of the cursor was ever within the target, the target was considered to have been hit. If the participant missed the target, the target continued to move along its path until it left the screen. If it had been jittering, it continued to jitter. If the participant hit the target, there was a sound and the target was displayed for 0.5 s at the position at which it had been hit, without any jitter. In contrast, if the cursor had been jittering, it continued to do so as it followed the finger until 0.5 s after the hit or 1.5 s from when the target appeared (whichever was first). Once it stopped jittering, the cursor was realigned with the finger.

#### Analysis

To answer our question, we needed to determine how the response to the steps constituting the jitter changed during the movement. We expected the vigour of the response to depend on how much time was available for adjusting the movement towards the target, so we needed to define the end of the movement in a way that reflects this remaining time, rather than the time until the hand stopped moving (which was usually well after crossing the target’s path). Moreover, we wanted to include trials in which the finger missed the target in our analysis. We therefore considered the moment that the finger crossed the path of the target’s centre to be the end of the movement. Occasionally, the finger did not move that far. In such cases, we considered the moment at which the finger was at its maximal distance from the starting point to be the end of the movement (as long as that was less than 2 cm from the target’s path). This allowed us to consider all but ten trials (in which the movement never came close enough to the target’s path). Image frames (and therefore steps) were selected from the end of the movement backwards in time. Since our analysis is concerned with responses to jitter in the presented images, trials were synchronised with respect to the times of these frames (presented every 17 ms; 60 Hz), rather than the ends of the movements themselves (determined to within 2 ms; 500 Hz). The last frame to be considered for the synchronisation was therefore 0–17 ms before the end of the movement.

The responses to the jitter are too small to relate fluctuations in individual finger movements to the jitter in single trials. To determine how each participant responded to steps at different moments, we averaged all his or her trials in which the step at each selected moment was to the left or to the right. Since the directions of the steps were completely independent of each other, the steps at all other moments than the one that was selected were as often to the left as to the right, so the average displacement at all other times is zero (Fig. 2C, D). We selected steps at all relevant times during the movement, and compared the average hand movements in trials in which there was a leftward step at that time with ones in which there was a rightward step at that time. Any systematic difference between these average trajectories was attributed to the direction of the selected step. We could do so, because the two average velocities each contained about as many responses to leftward as to rightward displacements at all other times. This procedure allowed us to use the same trials to quantify the responses to steps at all relevant moments, both for the target and the cursor (for a more detailed explanation of the method, see Brenner et al. 2023).

To quantify the response, we use a measure based on the lateral acceleration of the finger. For each trial, the lateral acceleration at various moments with respect to the moment of interest was determined by fitting a second-order polynomial to the measured lateral finger positions within a 40 ms window centred on the moment in question (simultaneous filtering and differentiation using a Savitzky–Golay filter). We determined the difference between the average lateral acceleration of the finger on trials in which the selected step was to the left and trials in which the selected step was to the right. This was determined for 250 ms from the time of the selected step. We considered a lateral acceleration to be positive if it compensated for the step: if the finger accelerated in the direction of the target step or in the opposite direction than the cursor step. Since it takes at least 100 ms to respond to a change in position, we only considered steps that were more than 100 ms before the end of the movement.

We assumed that, for the target, the vigour of the response would increase as the movement progresses, as was the case in our previous experiment using a different task and set-up (Brenner et al 2023). Besides checking this assumption, we examined whether the vigour of the response increases in a similar manner for the cursor. To do so, we plotted average responses for steps at different times, both for the target and the cursor. Moreover, to make it easier to evaluate whether the vigour of responses to cursor and target steps changes in the same way during the movement, or whether the vigour of the response is particularly weak early in the movement and particularly strong near the end of the movement, we plotted the value of the response to a cursor step as a function of the value of the response to a target step for various moments of the step. We did so for three times after the step. We reasoned that if the vigour of the response increases in the same way during the movement for the cursor as for the target, the points representing different moments of the step would lie on a straight line through the origin. If the vigour of the response increases faster towards the end of the movement for the cursor, the points would lie on a curve that bends upwards.

## Results

After adding six short sessions to compensate for data lost due to technical failure, and removing the 10 trials in which the finger did not move far enough, we had an average of 203 trials with target jitter and 202 trials with cursor jitter per participant. Of these, 70 ± 7% and 71 ± 6% were hit (mean ± standard deviation across participants). The end of the trial, as we defined it, was 580 ± 69 ms or 578 ± 70 ms after the target appeared (mean ± standard deviation of the participants’ mean values). Thus, we can compare the responses to steps of the target and cursor at different moments without having to worry about differences in performance or in overall time taken.

**Fig. 3:**
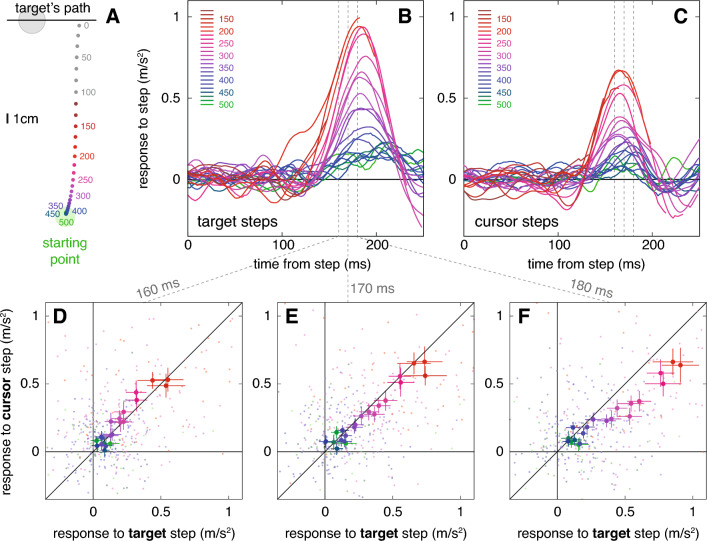
Responses to target or cursor steps at various moments. **A** Average cursor positions at the moments from the end of the trial at which consecutive frames were presented. The numbers indicate the corresponding times from the last frame. Note that not all points are numbered. The colour coding identifies the frame for which the step was considered for the curves and points in the other panels. The larger green and grey circles represent the starting position and the target. The horizontal line represents the target’s path. The next two panels show how the difference between an additional 2.5 mm target (**B**) or cursor (**C**) step to the left or to the right influences the lateral acceleration of the finger (for steps at different moments). If the selected step was less than 250 ms before the end of the trial, the curve stops at the end of the trial. Numbers and colours as in **A**. The remaining panels show direct comparisons of responses to target and cursor steps at three different times after the step: 160 ms (**D**), 170 ms (**E**), and 180 ms (**F**). Small points are individual participants’ values, several of which are not visible, because they are beyond the limits of the panel. Large points are means and standard errors across all participants

The average position of the finger at various times with respect to the end of the movement are shown in Fig. 3A. The dots’ colours indicate the remaining time to the end of the movement. We analysed responses to steps from before the finger started to move (green dots hidden below blue ones at the starting point) until the last moment at which we can expect any response to a target step (more than 100 ms before the end of the movement, so steps corresponding with the grey dots were not analysed).

As expected, responses were more vigorous for later target steps (red and purple curves above blue and green ones in Fig. 3B). They were also more vigorous for later cursor steps (Fig. 3C). Comparing the curves in Fig. 3B with those in Fig. 3C suggests that the responses to cursor steps start slightly sooner but last less long and are less vigorous than the responses to target steps. Consequently, the responses tend to be slightly larger for the cursor than for the target 160 ms after the step (many dots above line in Fig. 3D), but they are smaller 170 ms and 180 ms after the step (most dots below the line in Fig. 3E, F). Importantly, Fig. 3D–F shows that the responses to the target and cursor both depend on the time of the step in a very similar manner (they more or less fall on a straight line through zero). Thus, responses early in the movement were clearly less vigorous, but not disproportionally so for the cursor.

## Discussion

The aim of this study was to find out whether people rely more strongly on visual information about the position of the hand as it approaches the target. We found that the vigour of the response to perturbations increases in the same way during the movement when a cursor that follows the participant’s finger is perturbed as when the position of the target is perturbed (Fig. 3). Thus, participants do not rely on kinaesthetic information at the start of the movement, and gradually increase their reliance on visual information as the cursor approaches the target. Neither do they generally rely more on (visual) feedback late in the movement, as is often assumed (Elliott et al. 2017; Woodworth 1899).

We know that people respond more vigorously when there is less time left in which to make the required adjustment (Brenner et al. 2022, 2023; Oostwoud Wijdenes et al. 2011; Zhang et al. 2018), so an increase in response vigour on its own (Fig. 3C) does not prove that participants increase the extent to which they rely on visual information. That is why we examined whether the vigour of adjustments to cursor displacements depends on the remaining movement time in a clearly different manner than does the vigour of adjustments to target displacements. It does not: the overall vigour is different (Fig. 3B, C), but the vigour of responses to target and cursor jitter change in the same manner during the movement (shown for three moments at which both are relatively clear in Fig. 3D–F; the points form a more or less straight line through the origin, as expected if the response to cursor jitter is a constant fraction of the response to target jitter).

That the overall response to cursor jitter is less vigorous than that to target jitter is hardly surprising, because kinaesthetic information about the finger is not jittered, so also considering kinaesthetic information about the finger (Kasuga et al. 2022; Sober and Sabes 2005) should reduce the vigour of the response to jittering the cursor. In an experiment in which the cursor moved on a vertical screen, whilst the hand moved a mouse over a table, making it difficult to combine visual and kinaesthetic information about the cursor’s position (Kasuga et al. 2022), responses were equally vigorous for single steps of the target and cursor (Brenner and Smeets 2003). In that study, the response was also slightly earlier for the cursor, as it was in the present study, confirming that participants do not simply rely on the visual separation between cursor and target.

One might argue that the increase in the vigour of adjustments to cursor displacements later in the movement arises from relying more on visual (rather than kinaesthetic) information as the cursor approaches the fovea and can therefore be localised more precisely visually. However, this argument does not hold for the target, for which one must fully rely on vision throughout the movement. As the vigour of responses changes in a similar manner for the target and the cursor, it is unlikely that gradually shifting between sources of information plays an important role in regulating the vigour of the response to cursor jitter.

In our previous study with jittering targets (Brenner et al. 2023), the peak velocity of the response was usually well before 200 ms after the selected step. In the present study (Fig. 3B), the velocity of the response continued to increase (positive acceleration) until well after 200 ms. The latency of the response also looks slightly longer in the current experiment. These differences might be related to the friction that arises from sliding a finger over a table rather than moving it through the air, or they might be related to sliding one’s finger through the target rather than tapping on it (in line with the differences observed by Scheidt and Ghez 2007). However, there were many other differences between the studies, such as whether one was guiding a cursor or the hand itself, the orientation of the surface, the frame rate, and the target size, so we cannot be sure that the difference is due to the task.

As in earlier studies (most notably Saunders and Knill 2003, 2004, 2005), we studied the control of visual feedback about the hand by perturbing the position of a cursor that follows the hand. One could argue that visual information is likely to play a more important role when the hand itself is visible, because seeing the whole hand (and arm) provides more information than is provided by a single point representing the fingertip. On the other hand, one could argue that visual information is likely to play a more important role when only a cursor representing the hand is visible, because a cursor provides slightly delayed information about the position of the hand, so practise guiding a cursor to the target may discourage people from relying too heavily on kinaesthetic information when trying to hit the target with the cursor. Due to such considerations, we do not think that one should attach too much significance to the extent of the difference in vigour between responses to target and cursor steps in Fig. 3. Determining how visual and kinaesthetic information is combined to guide the moving hand would require a much more extensive study. Our main finding is that the vigour of responses to steps in the target and cursor position increased in a similar manner during the movement, as the available time decreased. This implies that people do not rely less on visual information about their hand than on visual information about the target early during the movement when visual information must be acquired through peripheral vision.

Finally, we would like to note that we analyse and interpret our results in terms of responses to steps. The terminology that we use suggests that there is a planned movement and that observing deviations from that movement (as a result of cursor steps) or evidence that the plan is incorrect (as a result of target steps) gives rise to corrective responses. This description matches the way responses to visual (Paulignan et al. 1991; Saunders and Knill 2005) and kinaesthetic (Crevecoeur et al. 2012) perturbations are described in the literature. However, for continuous responses of which the vigour is adjusted to the remaining time, it might be more fruitful to think of the movement as constantly being re-planned: at each instant, the brain figures out what muscle activations will move the finger to the target. This is a form of direct control (Zhao and Warren 2017), but direct control by a prediction based on the instantaneous information, rather than direct control by the information itself (Lee et al. 2001). Such control does not consider deviations from a former plan (errors) but simply re-plans the movement at each instant. The advantage of thinking about movements in this way is that changes in the environment (Crowe et al. 2023), changes in one’s own state due to external forces (Nashed et al. 2012), and choices between options (Brenner and Smeets 2022), can all be considered together at each moment.

In the present study, we show that the vigour of adjustments to cursor steps depends on the remaining movement time in the same way as does the vigour of adjustments to target displacements. This is inconsistent with the idea that early stages of the movement are ballistic, with visual guidance of the hand only emerging late during the movement (Elliott et al. 2017; Woodworth 1899). We propose that earlier studies misinterpreted the fact that the vigour of responses is weaker early in the movement, so that adjustments are less easy to detect, as evidence for such a distinction between different stages of the movement.

## Data Availability

The data and analysis programme are available at https://osf.io/347fa/?view_only=a0bf7d72b18144d1825b0353728b7e21.
